# A novel degradable PEG superparamagnetic iron oxide capsule coupled with a polyphenolic nano-enzymatic conjugate (PSPM-NE), to treat ROS-driven cardiovascular-diseases, tested in atherosclerosis as a model disease, and hypothesizing autoimmunity as an atheroma's trigger

**DOI:** 10.3389/fcvm.2024.1125571

**Published:** 2024-07-02

**Authors:** Glaucia C. Pereira

**Affiliations:** Department of Bioengineering, Faculty of Engineering, Imperial College London, London, United Kingdom

**Keywords:** reactive oxygen species (ROS) and nitric oxide (NO), chronic inflammation, atherosclerosis and auto-immunity, nanotechnology, polyphenols, nano-enzymes, magnetically driven nano-composites, the ThreeIB computational method

## Abstract

Cardiovascular diseases account for a significant portion of the worldwide mortality rate. This aroused interest among the specialised scientific community, seeking for solutions based on non-clinical and clinical investigations, to shed light onto the physio-pathology of cardiovascular impairment. It is proven challenging managing chronic cardiovascular illnesses like atherosclerosis, arrhythmias, and diverse cardiomyopathies. In certain cases, there is no approved treatment. In other cases, the need for combining therapeutic components, when dealing with co-morbidities, may increase the risk of toxicity-driven cardiovascular impairment. In this case, because the risk of cardiac events correlates with the QT prolongation rates, the QT or QTc interval prolongation has become an important biomarker to access drug-related cardio-toxicity. Several approaches have been found in the current literature, aiming at improving physiological acceptance, i.e., to reduce toxicity. Nanotechnology has increasingly appeared as a promising ally to modulate active substances, preserving cardiovascular function and optimising drug effectiveness, i.e., acting as a cardio-protective mechanism, leveraging the effects of drug-driven cardio-toxicity. In this manuscript, the author combines plant active compounds and nanotechnological strategies, e.g., nano-encapsulation, nano-enzymes, magnetically driven nano-delivery systems, applied in regenerative medicine, and assesses their effects on the cardiovascular system, e.g., as cardio-protective factors, reducing cardio-toxicity. The aim is to propose a new strategy to tackle atherosclerosis initiation and progression, in a drug design that targets ROS-removal and reduces inflammation, using auto-immunity biomarkers to select key atheroma-related signalling cascades. To analyse physiological phenomena related to atherosclerosis initiation and progression, the author proposes both experimental observations and a new haemorheological computational model of arterial constriction. The results of such analysis are used as motivators in the design of the here presented strategy to tackle atheroma. This novel design is based on degradable polyethylene glycol (PEG) superparamagnetic iron oxide capsule coupled with a polyphenolic nano-enzymatic conjugate (PSPM-NE).

## 1 Introduction

Nanotechnology has contributed translating scientific advances into new therapeutic and diagnostic tools, optimising patient care, and helping building new knowledge of the physio-pathology of cardiovascular impairment.

Cardiovascular diseases like atherosclerosis, arrhythmias, and cardiomyopathies can be initiated and modulated by diverse illnesses affecting other parts of the human body, including cancer, both bacterial and viral infections, along with drug-induced morbidity. Inflammation tends to play a key role, while drug-triggered toxicity may both initiate and aggravate cardiovascular conditions.

The assessment of drug-related cardio-toxicity is fundamental for drug safety. It is highly dependant of the analysis of cardiac physiological function impaired by drugs' dosage, during pharmacodynamic clinical trials. Nevertheless, early identification of the risk of cardio-toxicity, screening of patients' risk factors, and the development of preventive strategies in drug-induced toxicity are fundamental (1–6). The current literature has shown direct correlation between concomitant drugs and increasing risk of cardio-toxicity. Indeed, several works have shown an increase in risk of satellite metabolic impairment caused by drug concentration in plasma. Moreover, there is consensus in the fact that each concomitant drug alone poses a risk of cardio-toxicity that may be exacerbated in combination.

**Figure 1:**
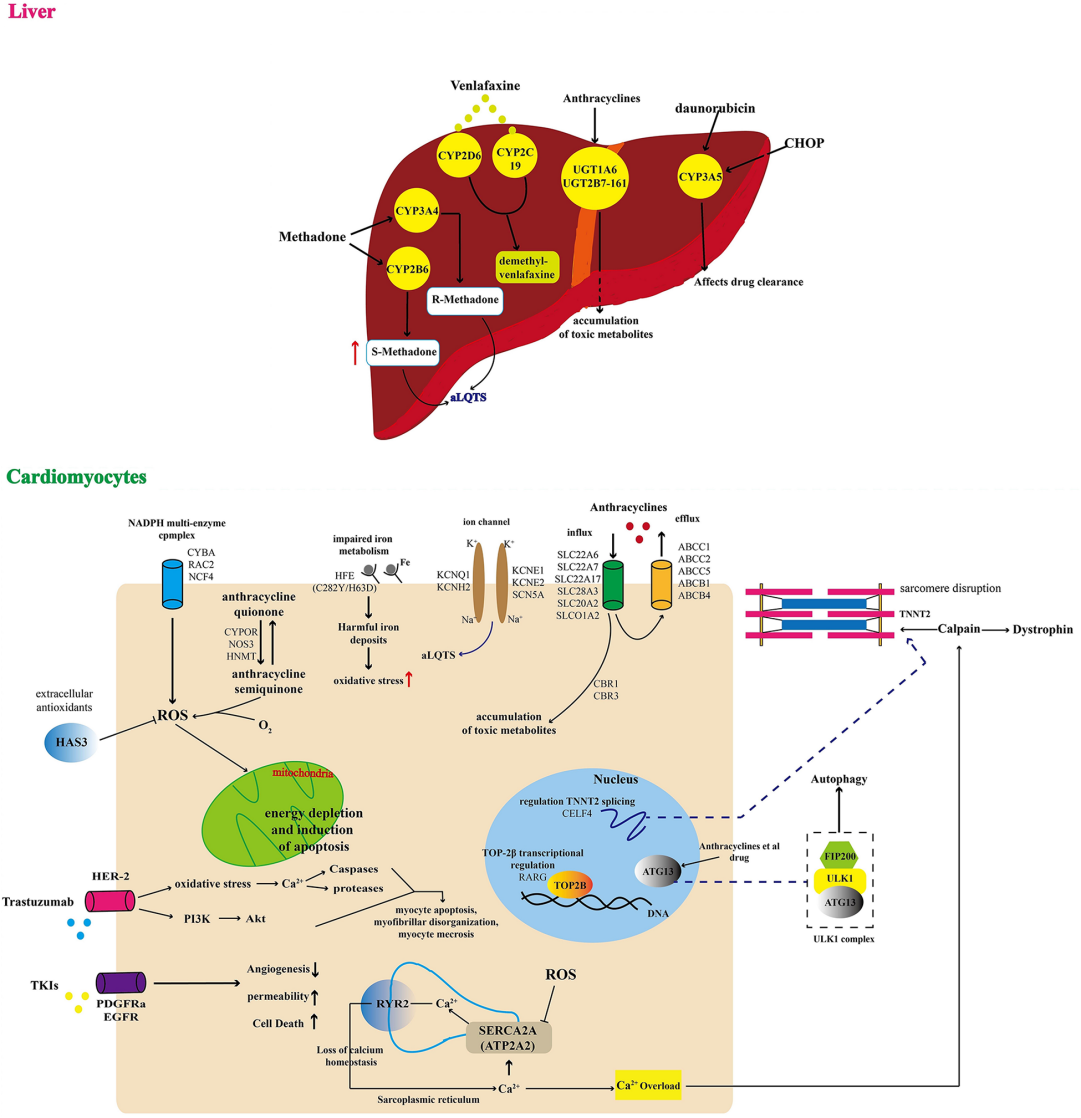
Schematic illustration of multiple genomic mechanisms of drug cardiotoxicity. (Liver) In the liver, QT syndrome due to drug toxicity is driven by multiple factors, including: (1) CYP3A5-driven disruption of drug clearance causing accumulation of compounds that in excess may affect other organs like the heart; (2) UGT1A6 and UGT2B7-161-driven accumulation of toxic metabolites that affect cardiovascular activity; and (3) increasing risk of acquired long QT syndrome (aLQTS), because of increases the plasma drug concentration. (Cardiomyocytes) In the cardiovascular system, QT syndrome may be directly induced by: (1) drug toxicity related energy depletion and induction of myocyte apoptosis, myofibrillar disorganisation, and myocyte necrosis; (2) disruption of calcium homeostasis affecting normal levels of angiogenesis, permeability, and cell death; (3) increasing risk of a damaging oxidative environment caused by multiple factors like impaired iron metabolism and ROS imbalance; (4) accumulation of toxic metabolites affecting cell nucleus and cell membrane ion-channels, resulting in increasing risk of aLQTS. Adapted with permission from (7), licensed under CC BY 4.0, https://doi.org/10.3389/FCVM.2022.966261/BIBTEX.

Recent studies have highlighted pharmacokinetics and pharmacogenomics as key elements in cardio-toxicity (7), as illustrated in Figure 1. Single nucleotide polymorphisms can modify the risk of toxicity. Therefore, designing genes encoding drug transporters, and optimising drug-driven enzymes' metabolism, could represent an enormous advancement in treatment design and dose selection. The transmembrane efflux of numerous cardio-toxic drugs is driven by drug transporters, e.g., anthracyclines. Gene polymorphism ATP-binding cassette family transporters may be targetted as biomarkers for cardio-toxicity, e.g., the P-glycoprotein ABCB1 complexes that alter protein folding can modulate drug-induced QT interval prolongation, which is an indicative of cardio-toxicity.

The prolongation of cardiac repolarisation (QT interval) can cause life-threatening polymorphic ventricular tachycardia (8–13). This phenomenon can be induced by drug toxicity (14). Early detection usually determines survival, because this phenomenon can be reversible, in early stages. Monitoring the QT interval requires expert medical intervention, because it is highly dependent of heart rate, some drugs, age, gender, and many other factors like sleep patterns (15).

In (16), an ECG-based calculation is presented, to manually measure and correct the QT interval.

In (17), the authors focused on tyrosine kinase inhibitors as key factors in the prolongation of the Bazet-based QT correction (QTc) interval, viewed on electrocardiograms (ECG). In that clinical study, the authors targetted sunitinib, vemurafenib, sorafenib, imatinib, and erlotinib, to ellucidate the role of tyrosine kinase inhibitors, in prolonged QTc intervals. They found a generally negligible QTc change. However, c. 21% of the patients assessed presented a clinically relevant QTc interval prolongation, equal or greater than 30 ms. Only about 5% of patients showed QTc interval prolongation equal or greater than 470 ms, which is associated with high risk of arrhythmias. However, their results were inconclusive, because it was unclear which risk factors would ultimately dictate QTc prolongation, i.e., age, low potassium, co-medication, and etcetera.

**Figure 2:**
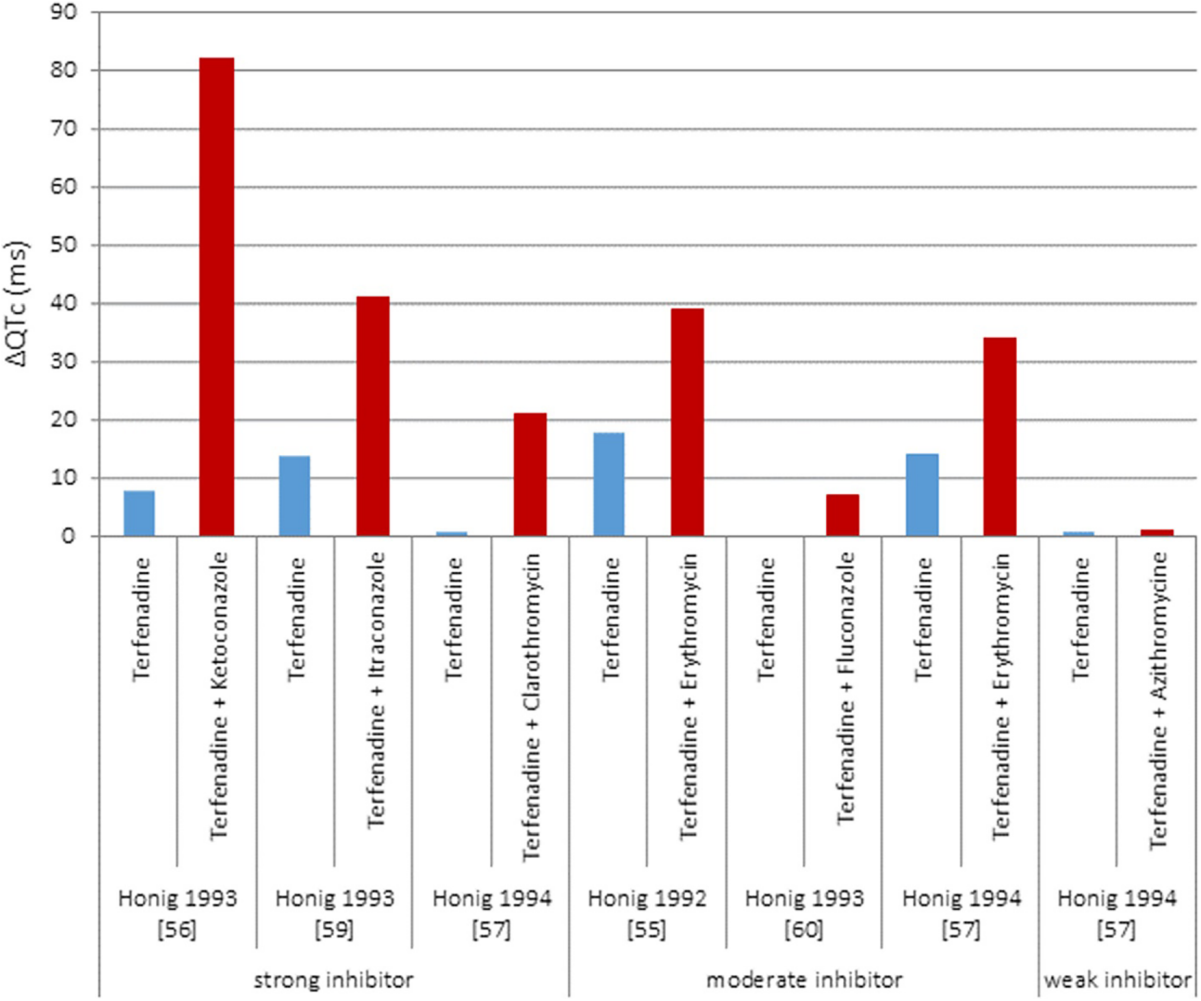
Results of five clinical studies with terfenadine given either alone or concomitantly with different CYP 3A4 inhibitors. Adapted with permission from (18), licensed under CC BY 4.0, https://doi.org/10.1186/S40360-016-0053-1.

Drug-drug interaction, i.e., concomitant drugs, is debated in (18). The authors found high variability in drug-drug interaction effects (Figure 2), e.g., they made reference to terfenadine-ketoconazole cases of cardiac arrest and pharmacokinetic drug interactions resulting in significant changes in blood or tissue concentrations driven by disruption of drug's absorption, distribution, metabolism, and excretion. Altogether, this makes understanding concomitant drug effects in QT interval challenging, which requires the assessment of myriad of risk factors, to better track drug-related electrophysiological effects, in clinical sceneries.

The QT interval is a very relevant indicator of drug-induced cardiac toxicity, which is usually demonstrated via a direct effect of the drug on ion channels. However, drug-induced toxicity can also result from phenotypic changes induced by repeated administration. These phenotypic changes could be detected via associated techniques, such as 3D echocardiography, MRI, and other imaging techniques currently used in patients.

How nanotechnology can mitigate key challenges in conventional therapeutics, including drug-induced toxicity? The opportunities are vast.

**Figure 3:**
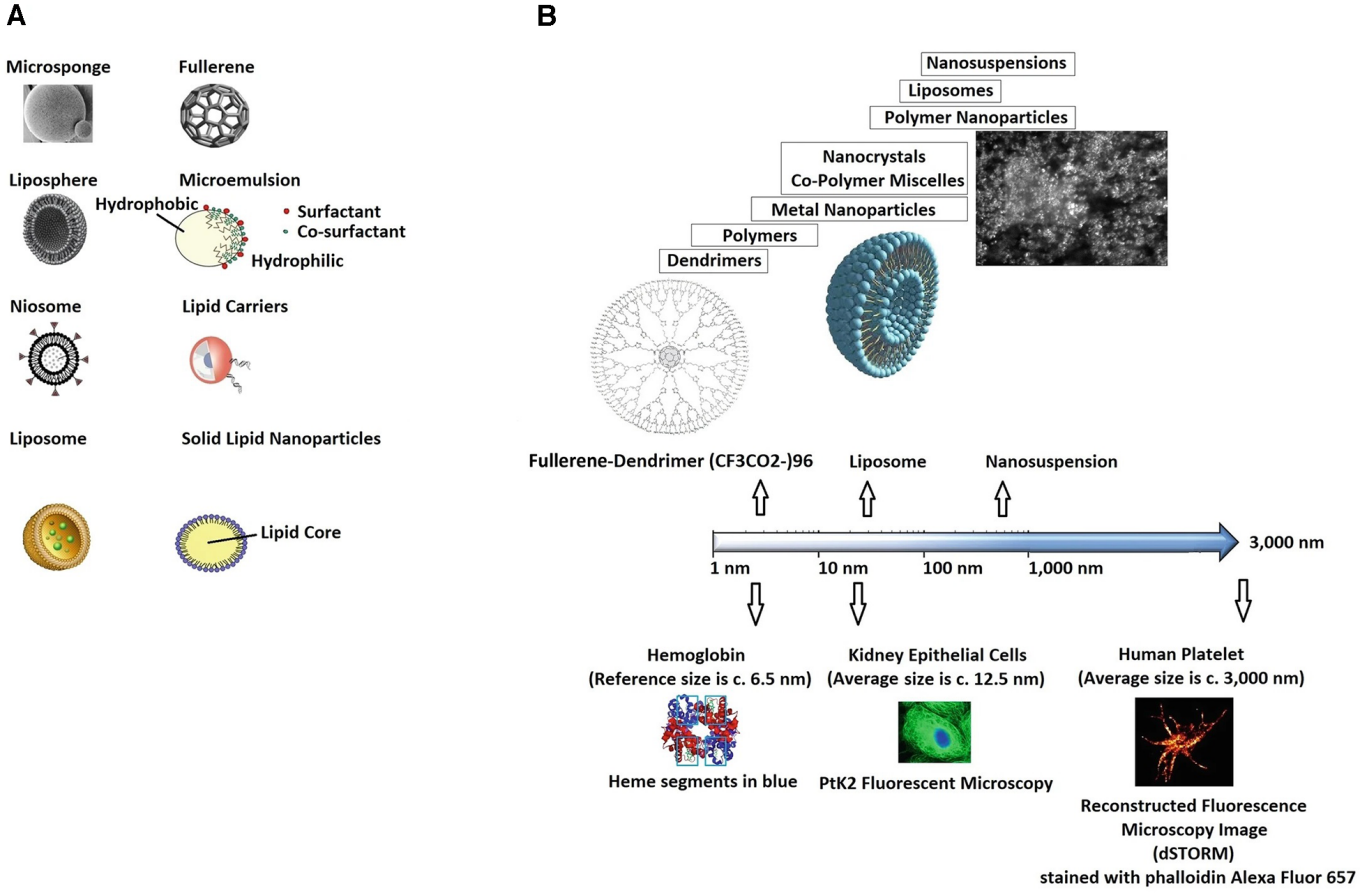
Illustration of (**A**) nanosystems, in their varied forms (carriers), and (**B**) reference scale. Adapted with permission from (19), Copyright © 2020 Springer Nature Singapore Pte Ltd, https://doi.org/10.1007/978-981-15-1761-7_24.

Evolving nanoparticles' technology and improved nanoscale systems' control, in precision medicine, have had significant impact on tissue engineering and regenerative medicine (19–24), Figure 3. Among the benefits of nano-complexes based therapeutics are low toxicity, optimised material properties (25–28), tailored pharmacokinetics and mechano-physics characteristics, targeted responses, improved delivery potential, e.g., via magnetically directed nano-composites (29), and better modulation-control over selected signalling cascades (30), e.g., by designing multi-modal nanoenzymes.

Nano-theranostics pursues the successful combination of low toxicity and high effectiveness in nano-diagnosis, monitoring, and therapeutics (21, 31), targetting simple and effective modes of bio-separation, imaging, and gene/drug delivery. The major goals in advancing nano-systems are to minimise the non-targetted interference of drug/monitoring carriers in proteins and cells' activity, sustain the medicinal properties of compounds after surface modifications, avoid elimination by the immune system (e.g., the blood-brain barrier), and reduce toxicity.

Either treating cardiovascular pathologies or other illnesses, nano-systems can significantly benefit patient-care, acting as cardio-protective agents (22, 32–37). In (38), the authors reviewed a set of commonly used nano-complexes, and their action in the cardiovascular network. In that review, the authors focused on the cardio-protective and regenerative capabilities of novel nano-materials. In (39), nano-materials were employed to treat neurodegenerative dysfunction. The authors discussed the benefits of nano-materials over conventional chemical or biological interventions, modulating misfolded proteins α-synuclein, amyloid-β, and tau signalling cascades, and mitigating oxidative stress effects, along with mitochondrial damage, inflammation, and cell death, to control neuro-impairment associated with Parkinson's and Alzheimer's disease. The approaches discussed are additionally beneficial to the cardiovascular system, by reducing the risk of toxicity. In (40), nanotechnology was discussed for increasing the efficacy of oncology-drug delivery, improving the design of medical compounds, to mitigate undesirable cardio-toxic effects. The authors highlighted the relevance of appropriate techniques for monitoring anti-tumor efficacy, assuring the preservation of the cardiovascular health.

In (22), the author discussed nanotechnology in precision medicine and inoculation protocols, designing nano-encapsulated mRNA vaccine, to leverage the burden of SAR-COV-2 pandemic.

Novel vaccine technology based on viral RNA is very susceptible to the surrounding environment (23, 41–44). In inoculation, the chemical instability of mRNA makes reaching appropriate manufacturing yields critical, because of the degradation of the *in vitro* transcribed products. Additionally, the resulting complexes are subject to restrictive storage conditions, to preserve mRNA viability. Naked mRNA complexes would be rapidly degraded by RNA's hydrolytic cleavage and attack by nucleases, oxidizers, and chemical modifiers. In the human body, mRNA exposure to Mg2 + and high physiological temperatures would equally cause mRNA instability. Remarkably, knowledge on nano-delivery systems is highly transferable to myriad of therapeutics.

Altogether, nanotechnology has emerged as an important co-adjuvant in numerous clinical studies, to increase drug effectiveness, decrease toxicity, better patient follow-up and monitoring, and to advance in early diagnosis.

In this manuscript, the author focus on low-toxicity drug design to treat atherosclerosis. First, selecting important signalling cascades, based on prior analysis of atheroma progression via both an haemorheological computational model of atheroma progression and experimental findings. Second, combining polyphenolic nano-enzymes and a magnetically driven nano-delivery system.

The novel method here discussed (i) describes the results of the author's haemorheological computational model for analysing phenomena in focal sites of interest and dysfunctional signalling cascades, (ii) uses those computational findings in combination with the author's experimental results on haemorheological flow under cell culture conditions, to motivate and propose therapeutic components as polyphenols, to mitigate dysfunction related to ROS imbalance and auto-imune factors, and (iii) presents nano-strategies for encapsulation and delivery of therapeutics at sites of interest, optimising results and mitigating side effects, in atheroma initiation and progression.

## 2 A synergistic combination of nanotechnology, results from computational design experimentally validated, and plant active compounds to mitigate cellular damage caused by reactive oxygen species (ROS), to treat atherosclerosis, under the perspective of auto-immunity

### 2.1 Results from an haemorheologic computational approach to analyse atheroma initiation and progression at predilection sites versus experimental microfluidics and cell culture

Chronic arterial inflammation is a strong biomarker in atherosclerosis. Atherosclerotic plaques cause the blockage of the arterial lumen, reducing blood supply to the body, and, in case of plaque rupture, they can result in clot formation and further blockage of blood flow to important organs, like the heart and the brain. Atherosclerosis and its satellite illnesses are among the main causes of global mortality (43). Fluid-mechanic forces are fundamental to atheroma formation. Shear stress is sensed by endothelial cells, triggering transduced chemical responses that regulate cellular activity. In (44), the author showed that changes in shear stress, i.e., changes in flow patterns caused by blood particles, can unveil new modifications in both the magnitude and the distribution of mechanical forces sensed by the endothelium. This, comparing the commonly used Newtonian models of blood flow in diseased arteries with a novel particulate blood flow model deriving from a new numerical method named the ThreeIB method (45). Observing these differences is particularly important, because designing new therapies for long-lasting diseases like atherosclerosis relies heavily on the computational simulation of phenomena that otherwise would take several years or decades to develop, or, whose nuances would never be fully observed in-vivo, nor in-vitro, again, because of the target time-frame. Hence, analysing blood flow characteristics under physiological conditions, using a computational framework, makes possible to design new effective solutions based on a better understanding of disease initiation and progression.

**Figure 4:**
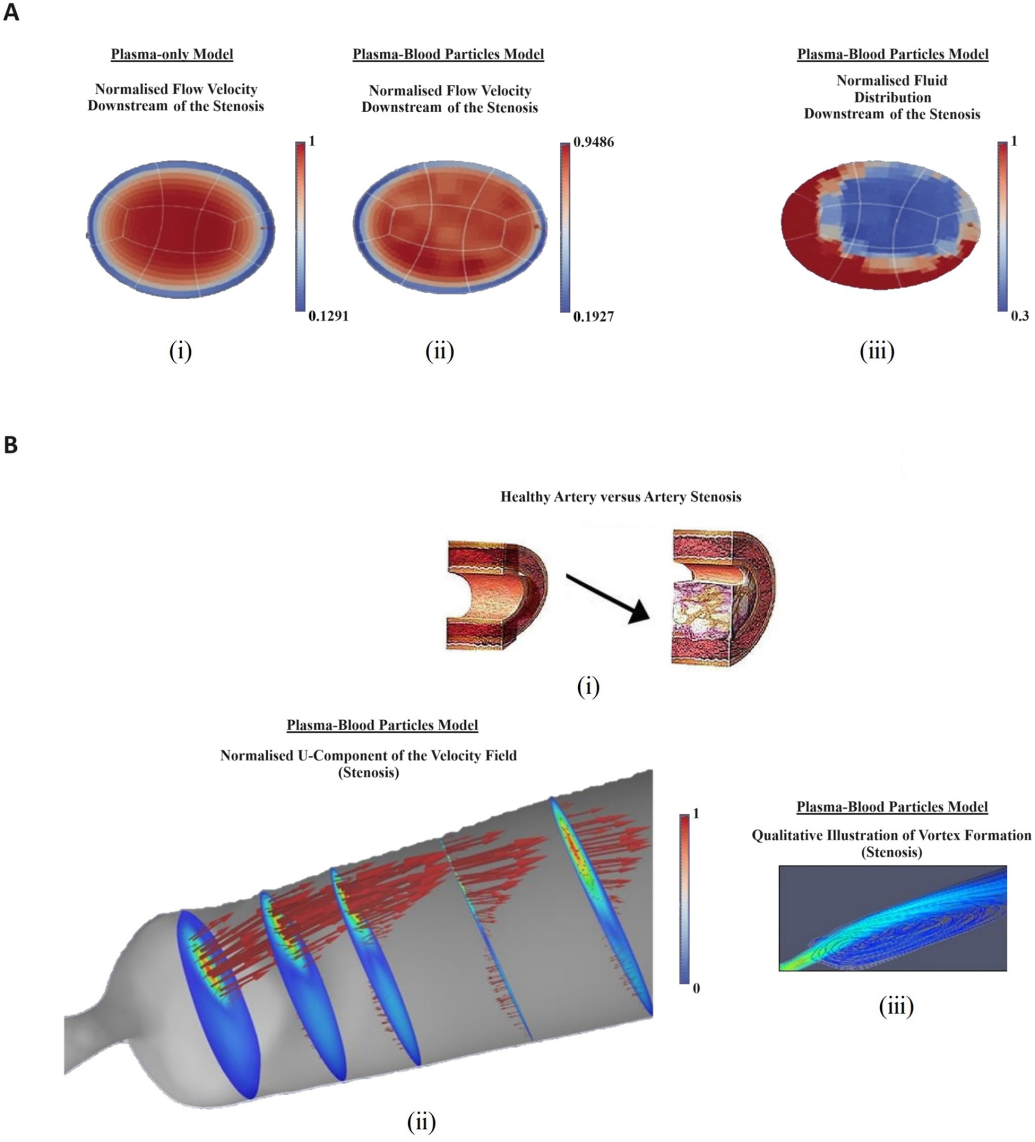

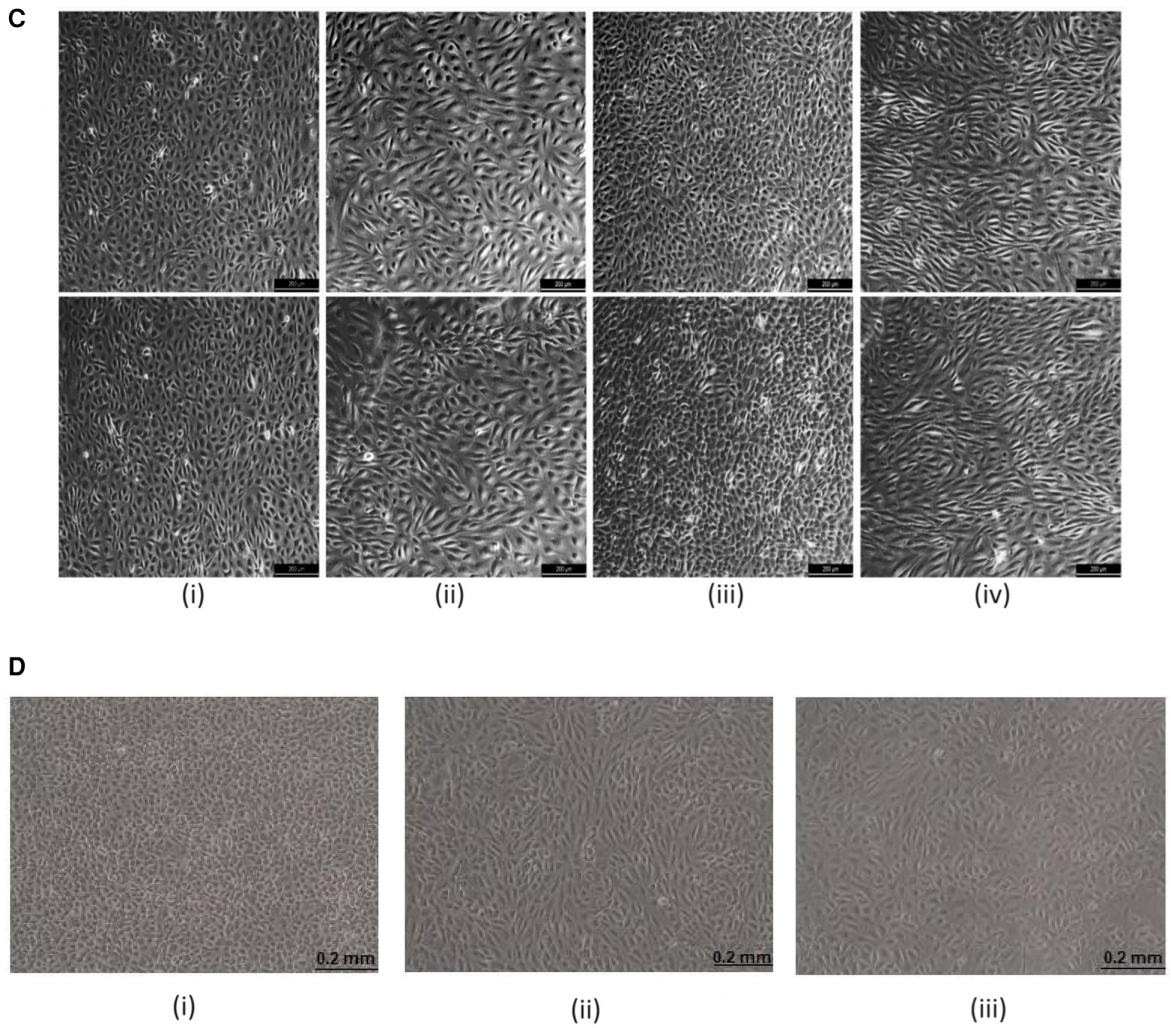
Computational and experimental results illustrating the relevance of haemorheology in studies of atherosclerotic plaque development: (**A**) computational rheological blood flow in a mouse carotid artery, at Re = 58: cross-sectional view of the stream-wise velocity. (**i**) normalised single-phase flow velocity (m/s velocity converted into 0–1 scale); (**ii**) normalised multiphase flow velocity; (**iii**) normalised fluid distribution at an arterial cross section (0%–100% converted into 0–1 scale); (**B**) Computational rheological blood flow in a mouse stenosis (carotid artery), with Re = 58: (**i**) illustration of lumen progressive narrowing caused by atheroma formation; (**ii**) normalised blood flow U-velocity at the stenosis with flow direction indicated by arrows and, (iii) stream-lines downstream of the stenosis, showing a region of re-circulation; (**C**) Experimental Results showing deleting shear stress alignment from primary endothelial cells: (**i**) cell cultured under static (no flow) condition without KLF2 down-regulation. The cell monolayer presents the endothelial specific cobblestone morphology; (**ii**) cells exposed to 15–20 dyne/cm2 shear stress generated by the flow of culture media for 24 h, without down-regulating KLF2 expression. The cells align following flow patterns resulting from a pulsatile inlet; (**iii**) cells electroporated with siRNA, which targets and down-regulates KLF2 expression. Subsequently, 15–20 dyne/cm2 shear stress generated by a pulsatile flow was applied to the cells for 24 h. KLF2 down-regulation with siRNA technology deleted the shear stress alignment functionality from the endothelial cells; (**iv**) cells electrophorated with siRNA which does not target any gene in the genome. Then, the cells were subjected to oscillatory shear stress for 24 h. The cells align indicating that the electroporation procedure does not significantly affect the cells properties; (**D**) Cell Alignment: EA.hy926 alignment under different flow conditions—bright-field images: (**i**) control-cells cultured under static conditions (no flow) for 6 h. The cell monolayer presents the endothelial specific cobblestone morphology; (**ii**) cells exposed to 0.5–1.0 Pa shear stress generated by the flow of culture media for 6 h, without erythrocytes. The cells align following pulsatile flow conditions; (**iii**) two-phase flow experiments (erythrocytes at about 45% haematocrit) in which the cells are exposed to 0.5–1.0 Pa pulsatile shear stress for 6 h. The contents of this figure were adapted from technical reports by GC Pereira (46)*, which were made available online in 2016, by the Icelandic Institute for Intelligent Machines. Lately, it was presented by GC Pereira in the Biotechnology World Convention 2016. New adaptations of the original contents of this figure were published by GC Pereira in collaboration with Z Kis in (47), licensed under CC BY, https://doi.org/10.4172/2475-7586.1000117 and by GC Pereira in (44), Copyright © 2017 Springer International Publishing AG, https://doi.org/10.1007/978-3-319-53880-8_12. *Note: It was first published online as original research by G.C. Pereira (a technical report) and made available at: https://www.iiim.is/wp/wp content/uploads/2014/05/GPereira2016JBiomedEngMedDevic.pdf (Accessed December 25, 2023), and then later published in collaboration with Z Kis as a review article, under the DOI: 10.4172/2475-7586.1000117.

To assess the effects of physiological haematocrit and arterial shape on blood flow patters that correlate with atheroma development, the author addressed the coupled effects of realistic arterial geometries (MRI and CT scans-based 3D reconstructed images) and non-Newtonian assumptions (44) (Figure 4), presenting experimental results to support the relevance of haemorheology in computational models of atheroma development. The resulting model emulated the effects of blood particles in diseased large arteries, because these are predilection sites for atheroma development. The author reproduced clinically relevant haemorheological flow.

Additionally, the author observed that although predilection sites for atheroma development have been fully identified, the reason why plaque develops around those regions is not fully understood (44), showing that simulating blood rheology, at those sites, could elucidate atheroma initiation. Indeed, in (48), the author discussed chronic inflammation, atheroma initiation, and biomarkers for autoimmune illnesses that may well correlate with the development of atherosclerosis.

In (44), Newtonian (plasma) blood flow simulations were validated by comparison with well-known results from the literature. Normalised pressure, velocity, and shear stress were captured at arterial segments where atheroma developed causing stenosis. Pressure dropped at the stenosis and increased downstream of the stenosis. The normalised centreline velocity peaked at the maximum constriction, negatively correlating with the normalised pressure.

Rheology patterns were introduced, to assess the effects of non-Newtonian blood flow on atheroma formation. The author found that rheology has a pronounced effect on blood flow characteristics. Indeed, pressure drop was attenuated in c. 30%, at the stenosis, because of flow deceleration caused by blood corpuscular compounds, with peak concentration of blood particles registered at the same site. Non-Newtonian peak velocity was c. 28% lower than the Newtonian one. This resulted in lowering blood viscosity, affecting recirculation, which developed much slower. Altogether, this modifies wall shear stress and apparent viscosity, indicating that Newtonian blood flow simulations exhibit recirculation patterns developing faster and viscous phenomena weakening. This results from the momentum interchange between blood particles and the flow (plasma), which is neglected in Newtonian approaches. The author showed that there is overwhelming evidence corroborating the notion that focal changes to the arterial lumen caused by plaque deposition modify flow features associated with plasma-particles interactions. Therefore, rheology models of blood flow seem to better reproduce physiology at sites of arterial narrowing caused by atheroma.

Flow characteristics have been widely reported as an important biomarker of chronic inflammation. Inflammatory signalling cascades are regulated by endothelial wall shear stress. Changes in either the intensity or the distribution of shear forces sensed by the endothelium might affect the mechanisms that drive inflammation, including shear stress-related ROS imbalance.

Atheroma formation is a focal phenomenon. Therefore, optimal therapeutics would rely heavily on the capability of targetting predilection sites for plaque deposition, the ability to successful analyse long-lasting phenomena (e.g., computationally) and the potential to deliver medicinal compounds and/or stem or genetic fragments-based regenerative complexes at those targeted locations. This remits us back to nanotechnology as delivery systems.

The author used the above discussed computational and experimental findings as motivators, when deciding which elements should be combined in a novel strategy to tackle atheroma initiation and development. Therefore, following both the computational and the experimental assessment of atherosclerosis trends, assuming that ROS-imbalance is a trigger for atheroma initiation and progression, by causing both mitochondrial DNA and protein alterations, resulting in dysfunctional apoptosis and inflammation, here, the author proposes magnetically driven nano-composites as co-adjuvants in the design of novel approaches to treat atherosclerosis, together with anti-oxidative plant active compounds.

### 2.2 ROS and inflammatory trends in atheroma initiation and development

In (48), the author highlighted focal high concentrations of LDL and increasing cell inter-facial gaps as promoters in endothelial wall LDL invasion. These phenomena are followed by ox-LDL formation, to trigger LDL removal. The author proposed an autoimmune explanation for atherosclerosis initiation, indicating immune impairment resulting in the imbalanced production of reactive oxygen species (ROS), abnormal cell apoptosis, and ROS driven DNA damage as key factors (49).

**Figure 5:**
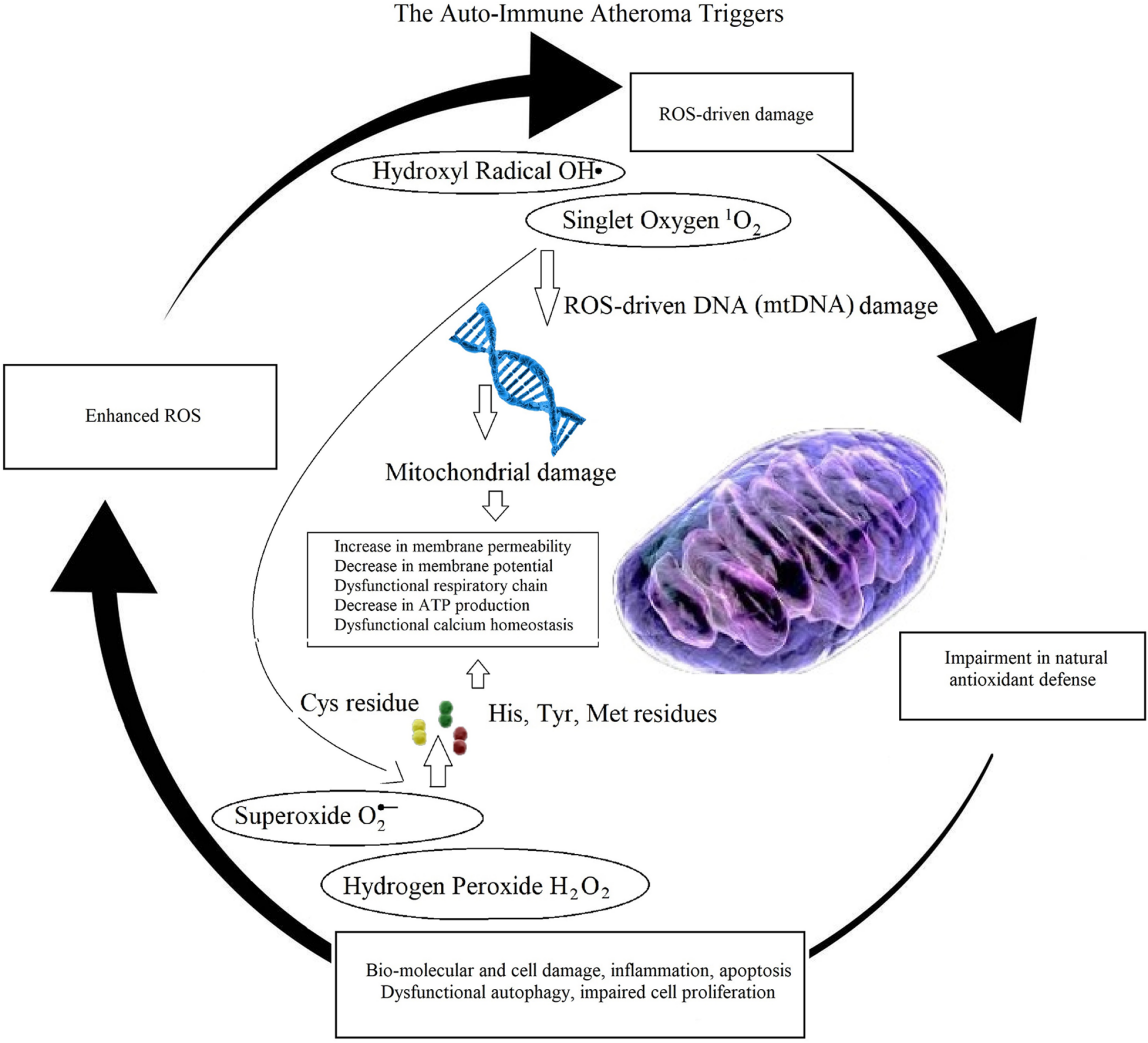
Illustration of auto-immune mechanisms in atheroma initiation and progression. A ROS-imbalance affects both DNA and intra-cellular protein translation, causing mitochondrial damages leading to apoptosis, inflammation, impairment in cellular autophagy and proliferation, negatively affecting natural anti-oxidative defenses, ultimately enhancing imbalance in ROS production, promoting cyclic oxidative cell and tissue deterioration.

The last is a well-known sign of autoimmunity related disease (Figure 5). Therefore, the author proposed that the abnormal concentration of ROS triggers atherosclerosis. The immune system contributes with cellular degradation. The author highlighted the role of oxidative stress and cellular dysfunction, in autoimmune pathologies as rheumatoid arthritis (RA), observing a similar pathological oxidative stress profiling in atheroma initiation. In autoimmunity, this oxidative stress profiling amplifies inflammatory responses, induces apoptotic cell death, and causes abnormal immunological activity. These findings support designing better therapeutics for atherosclerosis, to mitigate the abnormal distribution of ROS and their effects to the arterial wall.

In (48), the author showed that when cellular redox is altered at dysfunctional levels and mitochondrial function is affected, resulting in reducing ATP production, cell apoptosis can increase dramatically beyond the potential of cell proliferation. Here, the author hypothesises that, together with increasing concentration of LDL, at focal arterial locations, this phenomenon contributes to atheroma initiation and progression.

In (48), the author indicated that an imbalance between reactive oxygen species (ROS) production and their removal via protective mechanisms causes oxidative stress, which is among the major causes of inflammation-driven atheroma build up. Oxidative stress contributes with the activation of transcription factors that are involved in protein expression-driven inflammatory pathways. Inflammatory cytokines modulate repairing signalling cascades during infection and tissue dysfunction. However, defects in cytokines signalling may result in resident inflammation. Inflammatory signalling cascades contribute to atherosclerosis, myocardial infarct, kidney disease, glioblastoma, diabetic retinopathy, and several other diseases.

Atherosclerotic patients are particularly affected by cholesterol-rich oxidative plaque deposition. As indicated in former subsections, mechanical biomarkers as shear stress, blood particles, and the underlying blood flow patterns are fundamental to identify both focal locations and atheroma-related target signalling cascades. Inflammatory biomarkers like the interleukin families, the nuclear factor-kappa B (NfkB), and the signal transducer and activator of transcription 3 (STAT3) are important allies in the design of novel treatments for atherosclerosis, by refining the selection of signalling pathways in disease progression, giving insights on genotype-phenotype correlations to be targetted for atheroma treatment.

The author's experimental analysis and investigations on key biomarkers for atheroma initiation and progression served as motivators for the therapeutic design here presented. Therefore, in this study, the author proposes atheroma therapeutics to mitigate both inflammatory signaling cascades traditionally associated with atherosclerosis (e.g., interleukin families related signaling pathways) and auto-immune biomarkers (e.g., ROS-driven mtDNA alterations correlating with apoptosis at sites of high LDL concentration). The last is a ground-breaking proposal in the design of atherosclerosis therapeutics, because common approaches tend to focus solely on Inflammatory biomarkers like the interleukin families, the NfkB, and the STAT3, among others. A systematic review of the existing literature on the topic shows that this might be the very first time that atherosclerosis therapeutic design synergistically combines the elements proposed by the author, and is driven by biomarkers for auto-immunity.

### 2.3 Superparamagnetic delivery systems

Magnetic and superparamagnetic delivery systems have recently become very popular in biomedical research. These systems adopt conventional therapeutics with the capability of being minimally invasive introduced and driven within the human body, reaching deep tissue with minimal distress to the patient, and displaying negligible citotoxicity when compared with other therapeutics like chemotherapy in cancer treatment.

In (29), the author discussed numerous techniques enabling nanotechnology-driven directed vectors as an application of electromagnetism, in precision cancer therapeutics. The author discussed the main purpose of targeting specific groups of cells, optimising drug concentration at sites where tumour is forming, and minimising off-target interactions in healthy tissue. The author discussed both passive and active methods of targeting delivery, elucidating their advantages and limitations, where passive methods utilise the over-expression of biomarkers in diseased tissue to target specific drugs, and active methods physically guide therapeutics to regions of interest. The author focused on magnetically-driven particles' motion as this can be remotely controlled by external magnetic fields, making medical interventions minimally invasive, and optimising the benefits for the patients.

In (50), green synthetic modified iron oxide nanoparticles (IONPs) were synthesised with methanolic extracts of *Artemisia Persica*, loaded with cisplatin. The authors discussed the effects of synthesised IONPs, PEGylated IONPs, and cisplatin-loaded PEGylated IONPs on the MCF-7 cancer cell line, using the HEK293 normal cell line as control. The authors aim was to investigate the stability and bio-compatibility of those metal-based delivery platforms, concluding that the synthesised nanoparticles showed high bio-compatibility when in contact with CT-DNA, negligible cytotoxicity against KEK293, but significant cytotoxicity against MCF-7. Moreover, IONP complexed cisplatin was appointed as a good alternative for conventional chemotherapy, in breast cancer treatment. That study highlighted some of the main benefits of using metal-based nano-therapeutics, with special attention given to their magnetic characteristics as saturation, magnetic permeability, and the magnetic retention properties of dry nano-particles, and their benefits for delivery composites.

In (51), the authors approached the biomedical challenge imposed by the blood-brain barrier, as a fundamental part of effective treatments for brain disorders. In this context, the authors addressed superparamagnetic iron oxide nanoparticles (SPIONs) as promising carriers of therapeutics or as a therapeutic system themselves, because of their electromagnetic properties, biocompatibility and biodegradability, being capable of penetrating the blood-brain barrier. Therefore, the authors proposed to label therapeutic nano-scale systems using SPIONs to track them crossing the blood-brain barrier, using magnetic resonance imaging (MRI) technology.

In (52), the authors discussed the limitations of traditional anti-cancer agents, not being able to penetrate into hypoxic zones. Therefore, not being effective eliminating tumour cells in those areas, because these blocking cell division strategies do not reach the target cells. The authors reviewed magnetic nano-particles as drug delivery systems, driven by external magnetic fields, because of their penetration potential and ability to tailor their properties for specific biological applications.

In (53), captopril-coated magnetic nano-particles were used as a dual-mode agent, i.e., for simultaneous MRI contrast and drug delivery. According to the authors, as a contrast, these particles resulted in darker MRI images. The authors reported on captopril release from γ-Fe2O3@SiO2@captopril being greater at pH 1.2, in comparison with drug release values at pH 4.8 and 7.4. Overall, the authors concluded that this composite showed acceptable characteristics to be used as a dual-mode agent.

In (54), the authors discussed superparamagnetic iron oxide nanoparticles as magnetically driven carriers, and their benefits over other nanoparticles-based systems. The authors focused on bio-compatibility and biodegradability, and the advantage of superparamagnetic iron oxide nanoparticles being driven by external magnetic fields, in a minimally invasive fashion. The authors reviewed numerous superparamagnetic iron oxide nanoparticle formulations, e.g., micelles composites, clusters, hydrogels, liposomes and micro/nanospheres, and assessed their potential delivering bio-therapeutics, e.g., cells, proteins and genes. The authors concluded that superparamagnetic iron oxide nanoparticles exhibit superior biocompatibility and superparamagnetic capabilities, enabling long-term accumulation at target sites. Because knowledge of their synthesis is well developed, these nano-systems are very attractive for clinical applications.

In (55), the authors reviewed optimisation strategies in sorafenib delivery into hepato carcinoma cells. Sorafenib is thought to be efficient containing hepato-carcinoma formation. However, it exhibits severe side effects, including high toxicity. Superparamagnetic iron oxide nanoparticles were proposed to deliver orafenib at specific sites, reducing off-target interactions in healthy tissue, and improving intra-tumour drug concentration. In that study, human hepato-cellular carcinoma (HepG2) cells were used as an *in vitro* benchmark, to evaluate the performance of the designed orafenib superparamagnetic iron oxide nanoparticles. The authors concluded that the designed delivery system enhanced sorafenib's anti-tumor efficacy, by targeting specific hepatic tumor sites, with remarkable selectivity, mitigating the reported side effects attributed to sorafenib alone.

In (56), a supraparamagnetic graphene oxide (GO/Fe3O4) hybrid nanocomposite was designed for drug delivery. The authors selection on GO/Fe3O4 assured high thermal conductivity and preservation of the loaded doxorubicin's anti-tumor efficacy. The authors assessed the potential cardio-toxicity effects inherent of the designed nano-composite, while testing its efficacy in cancer therapeutics, using the ehrlich ascites carcinoma breast cancer cell line. The results suggested high loading capacity surface area reaching up to 90%, low cardio-toxicity, and strong anti-tumor outcomes, but exhibiting a different therapeutic effect on cell cycle and apoptosis to that of doxorubicin alone. Overall, the hybrid form GO/Fe3O4 doxorubicin with folic acid, associated with brief hyperthermia, induced anti-tumor effects with reduced cardio-toxicity.

As seen in the above examples from the current literature, the application of magnetic fields to produce directed motion in biological tissue, either by attraction or swimming mechanisms has its advantages and limitations. As delivery systems, the current literature has increasingly shown that magnetic and superparamagnetic particles can well enhance drug, cell, and genomics-based therapeutics, remarkably mitigating side effects associated with conventional clinical strategies. However, the success of magnetically targeted delivery systems is still challenging. These delivery systems can be designed via non-toxic bio-compatible biodegradable materials. However, care must be exercised when defining disposal routes. In (29), the author highlights the need to assess potential long term drawbacks if these nano-composites remain in the body (e.g., if they would affect other medical procedures like MRI scans), their life-span, and the time it would take for them to be naturally excreted, otherwise. Additionally, utilising these magnetically-driven nano-particles in the human body, to treat atherosclerosis, would be highly dependent on local conditions, e.g., blood flow rate in large arteries where atheroma develops, because if the magnetic propulsion is not properly driven due to flow conditions and tissue characteristics, the delivery system would not serve its purpose.

### 2.4 The design of ROS and inflammation-based nanotechnological therapeutics for atherosclerosis

#### 2.4.1 Encapsulated plant active compounds

Among the varied compounds with a potential in innovative therapeutic design, for chronic inflammation and impaired ROS production, polyphenols are the ones that have been widely discussed in the literature (57–67). Polyphenols help inhibiting oxidases, supporting managing ROS at homeostatic levels, mitigating oxLDL formation, and modulating mitochondrial oxidative stress. This targets polyphenols as ideal ingredients to treat inflammation and defective cell apoptosis, which is fundamental in atherosclerosis initiation and progression.

In (48), the author discussed polyphenols treating autoimmune diseases and chronic inflammation, mitigating side effects like toxicity.

In this context, polyphenols are anti-oxidative-rich compounds with high potential to mitigate initiation and development of atheroma. Polyphenols help removing free oxygen and nitrogen species, mitigating defective pro-inflammatory activity like the imbalanced lipoxygenase (LOX) pathways, regulating inducible nitric oxide synthase (iNOS), and acting as vasodilators that reduce the risk of satellite cardiovascular illnesses.

In (19, 20, 22, 23), the author discussed nanotechnology, including nano-particles as novel delivery systems, to optimise administration routes. The motivation is to reduce cytotoxicity and increase biocompatibility of therapeutics based on encapsulation via nanoparticles, as polysaccharides.

In (68), dried aqueous-methanolic extracts from A. millefolium and lecithin were combined to obtain phenolic rich nanoliposome. The authors reported on moderate colloidal dispersion stability of the selected nano-complex, establishment of a homogeneous spherical shape, and homogeneous distribution of the phenolic compounds. Regarding the nano-complex potential for treating oxidative stress-driven inflammation, the authors concluded that the administration of the nanoliposome-encapsulated phenolic complex in mice models of liver disease resulted in significant reduction of both liver enzymes and lipid peroxidation, which are biomarkers for inflammation and oxidative stress.

In (69), a mouse model of high cholesterol showed that the phenol AGI-1067 is promising leveraging atheroma formation, by lowering LDL concentration in plasma, down-regulating mRNA-linked pro-inflammatory vascular cell adhesion molecule-1 and monocyte chemoattractant protein-1, and inhibiting TNF-α induction of redox-sensitive inflammatory proteins, vascular cell adhesion molecule-1, and monocyte chemo-attractant protein-1, in cell culture. This compound has shown significant anti-oxidant properties (70), indeed. The authors argued that, in humans, probucol derivatives are not shown to prolong the QT interval. However, their mouse model suffered from a prolonged QT interval that may induce cardiac arrhythmia. They found that a similar phenomenon was not observed in the administration of AGI-1067, concluding by proposing clinical trials to assess the safe administration of AGI-1067 in patients.

The administration of AGI-1067 in nano-complexes may mitigate potential side effects and potentialise drug administration at focal locations.

In (71), the phenolic phytochemical ferulic acid was used in poly(anhydride-ester) nanoparticles-based therapeutics to reduce inflammation in atheroma formation, by modulating foam cell formation and ROS imbalance, regulating macrophage lipogenesis.

Therefore, here, the author proposes plant active compounds combined with nanotechnology, because this combination represents a great alternative in the treatment of atherosclerosis, with special attention given to ROS imbalance and oxidative stress-driven cellular dysfunction.

#### 2.4.2 Nano-enzymes

Nano-enzymes are nano-materials with characteristics that aim at mimicking enzymatic activity. Among the most commonly used nano-enzymes are metal particles like Au, Ag, Pt, Pd, Ir, and IO@CO, metal compounds like MnO 2, CuS, and MnSe, non-metals like C-dots and fullerene, and non-metal compounds like g-C 3 N 4.

These materials have shown high applicability in clinics, with a remarkable potential for ROS-regulation. Therefore, the author here proposes these new therapeutics as part of the envisioned synergistic approach to treat atherosclerosis.

In (72), nanoenzymes were discussed as modulators of the ROS production, mitigating inflammation, including inflammatory processes related to auto-immune diseases like the inflammatory bowel disease. Nanoenzymes were discussed as an alternative to conventional approaches, whose side effects vary from antibiotics resistance to cardio-toxicity and renal dysfunction. Selenium nanoparticles were described as effective ROS-regulating agents, modified by Ulva lactuca polysaccharide, to improve the stability of Selenium. Additionally, the authors referenced the literature on Kluyveromyces lactis GG799 nanoparticles, suggesting that Ulva lactuca polysaccharide and Kluyveromyces lactis GG799 based Selenium nanoparticles performed well reducing oxidative stress and the underlying inflammatory responses. These nano-enzymes were promising in ROS-related inflammatory bowel disease, because of their effectiveness as ROS-scavengers. Similarly, in (73), ultra-small Rhodium nano-dots coated with polyethylene glycol were applied in the treatment of chronic inflammation during tumour formation, because of their ROS removal capabilities.

In (74), the authors assessed the ROS-regulating efficacy of iron oxide/cerium core–shell (IO@CO) nano-particles. Cell culture was targeted, to detect iron and cerium contents in macrophages. Cytotoxicity experiments were performed, treating macrophage J774A.1 cell cultures with ethidium homodimer-1 fluorescence dye, to detect dead macrophages. Macrophage J774A.1 cells were also used to assess intra-cellular ROS levels.

When cerium oxidation state changes between trivalent and tetravalent, cerium oxide can remove H2O2 and superoxide radical. ABTS [2,2′-azino-bis(3-ethylbenzothiazoline-6-sulphonic acid)] was oxidized by hydrogen peroxide (H2O2) in the presence of horseradish peroxidase (HRP) as a catalyst. The nanoparticles were incubated with hydrogen peroxide and HRP was added to the reaction. ABTS was introduced, to detect ROS. The authors reported on substantial absorbance reduction directly proportional to ROS levels, at cerium concentrations of c. 280 ng/100 μl and above. High concentrations of cerium in nano-complexes (e.g., 1,120 ng/100 μl) removed nearly half of the ROS in the solution. They concluded that IO@CO nanoparticles have a significant ROS-regulating ability, in buffer solution.

In (75), hyaluronic acid shell-based coated MnCO3 and MnO2 core particles of c. 1 μm diameter were assessed for toxicity and ROS-removal capability. The authors reported no toxicity, after 72 h of observations, and a high scavenging ability towards H2O2, along with effective modulation of O2 production. The core-shell particles exhibited efficacy in H2O2 removal, which is an important physiological waste in inflamed tissue, noting that 50 μg/ml of core-shell particles reduced the concentration of H2O2 from 1,000 μM to 60 μM. With optimal dose-dependent results achieved at 250 μg/ml of particles. Ultimately, an animal ROS model was used to assess citotoxicity. The results suggested that the used core-shell particles are not toxic to cells. A negligible reduction in metabolic activity was observed at particle concentration of 250 μg/ml.

Although showing promising properties as higher biocompatibility, potential to be magnetically driven to focal locations (metal-conjugates), and a multi-modal nanoenzymatic potential for treating ROS-combined-inflammatory cascades, in atheroma formation, the coupling polyphenolic nanoenzymes haven’t been widely utilised in the current literature.

In (76), a glucose oxidase-integrated metal-polyphenolic network as an activated cascade nanozyme was applied in hyperglycemic wound disinfection. The method was assessed resolving the difficulties in disinfection and healing of infected wounds, in patients with hyperglycemia. GOx-GA-Fe nanozyme was designed to exploit ROS generation for hyperglycemic wound disinfection, because of GGFzyme glucose's removal capability and high catalytic antibacterial capacity.

In (77), a tannin coordinated nanozyme composite-based hybrid hydrogel was employed for treating multidrug-resistant pseudomonas aeruginosa keratitis.

Fenton-derived therapeutics were used in (78), to degrade cancer cells, via reactive oxygen species obtained from endogenous H2O2 species. In that study, Cisplatin was designed as an artificial enzyme, to produce H2O2 for cellular apoptosis, modulating ROS-cascade reactions to optimise anti-tumor outcomes. The authors used epigallocatechin-3-gallate (EGCG), phenolic platinum(IV) prodrug (Pt-OH), and polyphenol modified block copolymer (PEG-b-PPOH), because of their high stability, versatile metal-polyphenol coordination interactions, and efficient intra-cellular drug release. Cisplatin catalyzed by an iron-based Fenton reaction optimally modulated ROS production. The authors reported improved results when comparing this approach with conventional chemotherapy. Moreover, systemic toxicity caused by platinum-based drugs was overcome.

In (79), the oxidation of phenolic compounds into highly reactive quinones was catalysed by polyphenol oxidase nano-enzymes (PPO), and PPO-silenced transgenic walnut lines were enginered, to study the PPO role in apoptosis. The authors compared the leaf transcriptomes and metabolomes of wild-type and PPO-silenced plants, concluding that PPO-silenced plants displayed greater alterations in the metabolism of phenolic compounds, their derivatives, and in the expression of phenylpropanoid pathway genes. The authors associated PPO types with the metabolism of tyrosine, with the biosynthesis of hydroxycoumarin esculetin, and with a significant increase in tyrosine-derived metabolite tyramine, which is highly related to apoptosis in walnut and several other plant species.

In (80), chronic inflammation and imbalance in ROS production in acute lung injury were treated with Fe-curcumin nanoenzymes. The Fe-curcumin complex triggered intra-cellular Ca2 +** **release, inhibited NLRP3-driven inflammation, and suppressed NF-κB activity, significantly contributing with intra-cellular ROS removal and reduction of inflammatory content. The authors reported reduction in macrophages differentiation (CD11b^lo^F4/80^hi^) and CD3^+^CD45^+^ T cells, in mice.

In Ultra-small natural product based coordination polymer nanodots for acute kidney injury relief (81), the joint plants' natural antioxidants and iron (Fe) ions were discussed, in the design of ultra-small coordination polymer nano-dots (CPNs), to harvest ROS in acute kidney injuries. The authors highlighted Fe-curcumin CPNs (Fe-Cur CPNs) role in ROS-removal and rhabdomyolysis-induced therapeutics.

Altogether, these are promising results, if translated into atherosclerosis' anti-inflammation nano-drugs. Therefore, novel approaches that benefit from polyphenolic nanoenzymes complexes' multi-modal characteristics would represent next generation therapeutics for ROS-based atheroma treatment.

#### 2.4.3 The proposed polyphenolic superparamagnetic nano-enzymatic conjugate named PSPM-NE

Proteins classified as enzymes are either difficult or expensive to produce and store. They are difficult to be designed in a stable form, and some times incompatible with environmental limitations, with several steric and catalytic challenges faced by existing protein engineering strategies. Therefore, using alternative compounds displaying similar catalytic activities has become an attractive solution.

To treat atherosclerosis, here, the author proposes the use of a nano-enzymatic conjugate combining SIRT-1 to stimulate mitochondrial biogenesis, Bcl-2 to attenuate apoptosis, UCP2 to remove ROS, and AMPK to optimise autophagy. This nano-enzymatic complex has the potential to attenuate inter-cellular gaps, modulate ROS production, and stimulate mitochondrial function and ATP production, corroborating with the maintenance of a health endothelium, free of inflammation-driven atherosclerotic plaque deposition.

**Figure 6:**
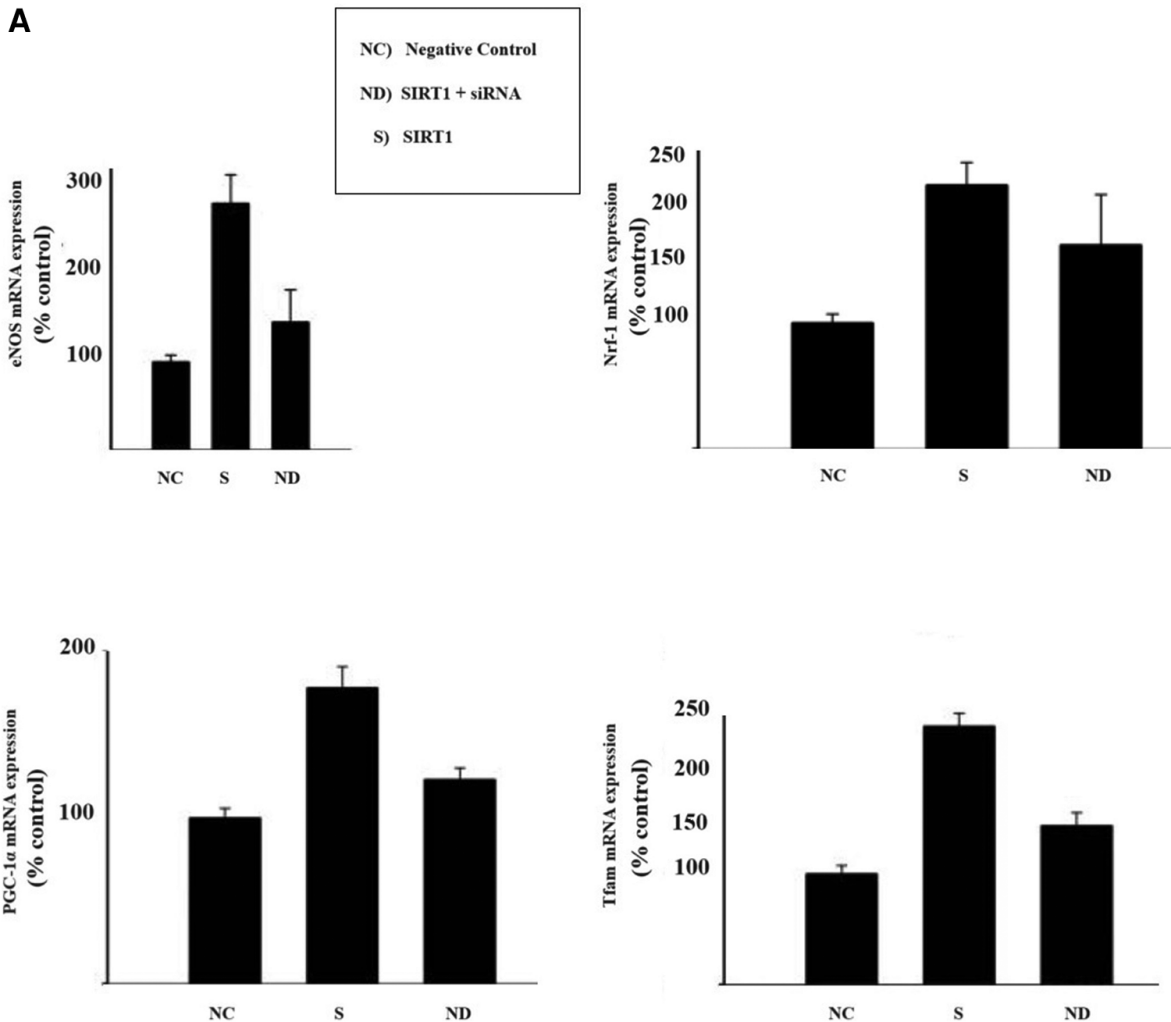

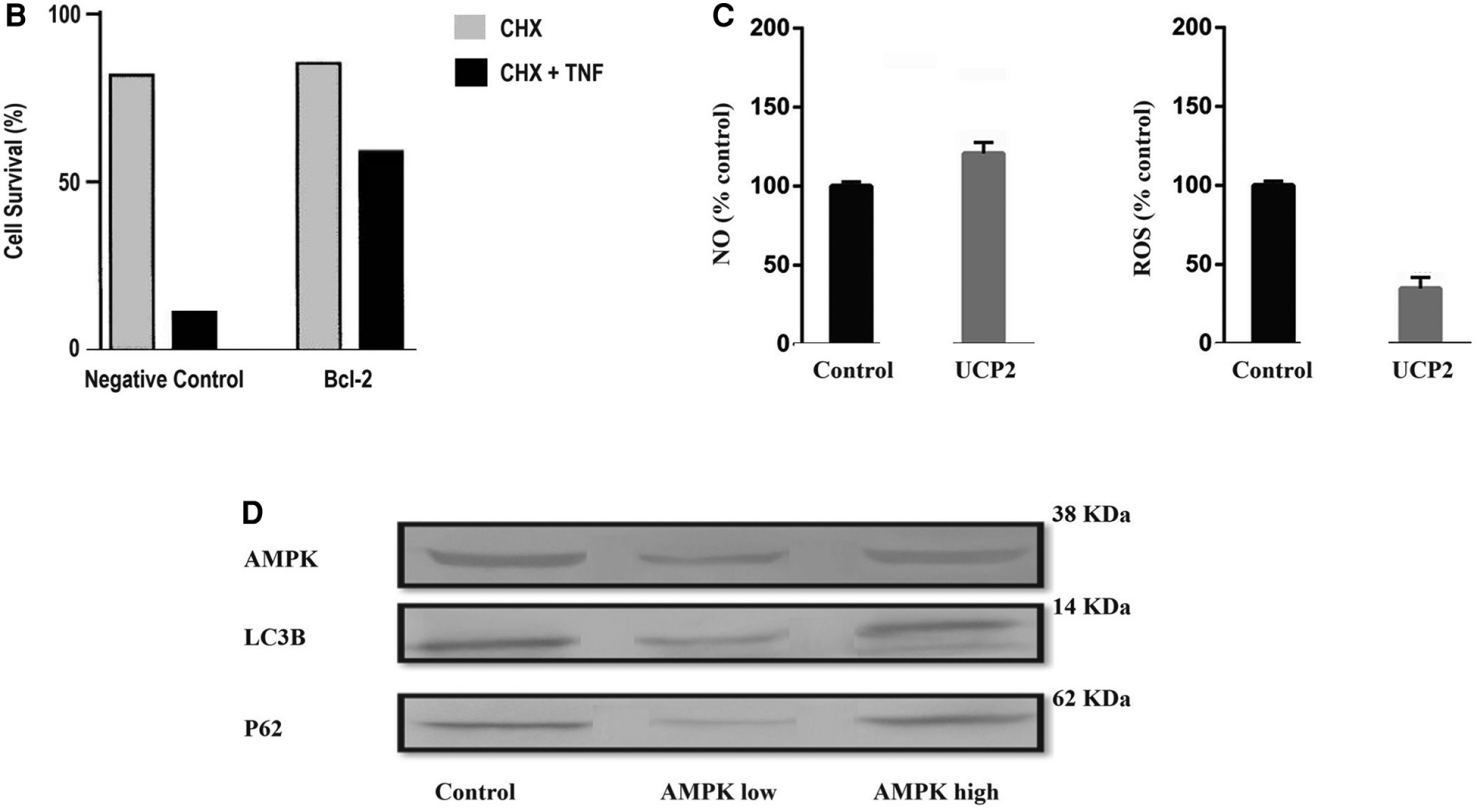
Illustration of the results of gene-expression in EA.hy926 cell culture: (**A**) illustration of mitochondrial biogenesis stimulated by deacetylase sirtuin-1 (SIRT-1), in a signalling pathway linked with the upregulation of endothelial nitric oxide synthase (eNOS). In presence of SIRT-1, endothelial nitric oxide (NO) synthase (eNOS) was upregulated (SIRT1-dependent) and knockdown of SIRT1 via small interfering RNA (siRNA) down-regulated eNOS. SIRT1 upregulated expression of PGC-1α and Tfam. Results for Nrf-1 upregulation were inconclusive. (**B**) Illustration of cell apoptosis attenuated by Bcl-2. Endothelial cells exposed to cycloheximide (CHX) become highly susceptible to tumor necrosis factor (NFT)-induced lysis. However, in presence of Bcl-2, NTF-mediated apoptosis effects dramatically decrease. (**C**) Illustration of Uncoupling Protein 2 (UCP2) potential reducing overexpression of oxidative species. It was observed a discrete increase in nitric oxide (NO) production and a reduction in reactive oxigen species (ROS) generation. (**D**) Western blot bands of AMPK, LC3B and P62. It was observed that the concentration of AMPK modulates the expression of the authophagy markers LC3B and P62. The cells were cultured until they reached 80% confluence. Overexpression of target markers were ascertained in cells for 6 h, before each experimental observation. The results are represented in percentage of the control (% control). Cell survival is represented in percentage of the initial cell count (%).

To motivate further research, the author shows in Figure 6 initial results with insights of the potential of this newly proposed therapeutic composite (PSPM-NE).

PSPM-NE seems to have the potential to be further investigated as an interesting option to improve arterial health, via the stimulation of mitochondrial biogenesis (expression of genes that sustain a healthier endothelium), via the reduction of NFT-mediated apoptosis (cell death) and the extenuation of the negative effects of oxidative stress, and by optimising cell renewal.

Remarkably, nanotechnology is embraced as an allay in precision medicine. Therefore, the designed nano-enzymatic conjugate is enriched by magnetic particles, to empower the final complex with the capability of being magnetically driven to focal sites of atheroma deposition.

Magnetically driven therapeutic elements are extremely versatile, because of the possibility of targeting specific areas of the human body, optimising drug-delivery routes and drug concentration at sites of diseased tissue, maximising drug utilisation and minimising the risk of toxicity. This, with the great advantage that magnetic fields penetrate the body with minimal attenuation and automate the delivery route via controlled particles' motion.

Therefore, to complete the design, the author proposes a degradable polyethylene glycol superparamagnetic iron oxide capsule that couples with the polyphenolic nano-enzymatic conjugate, forming a polyphenolic superparamagnetic nano-enzymatic (PSPM-NE) conjugate.

The PSPM-NE conjugate has the advantage of being fully degradable, bio-compatible, and is introduced in the human body in a minimally invasive manner, showing negligible cytotoxicity.

The PSPM-NE conjugate is directed to sites of atherosclerotic plaque deposition via pulsed magnetic field gradients. Magnetic resonance guides the PSPM-NE conjugate in the bloodstream to targeted endothelial segments. It is, indeed, widely evidenced that clinical MRI scanners can track the location of magnetically labelled particles, maneuvering them within targeted tissue.

**Figure 7:**
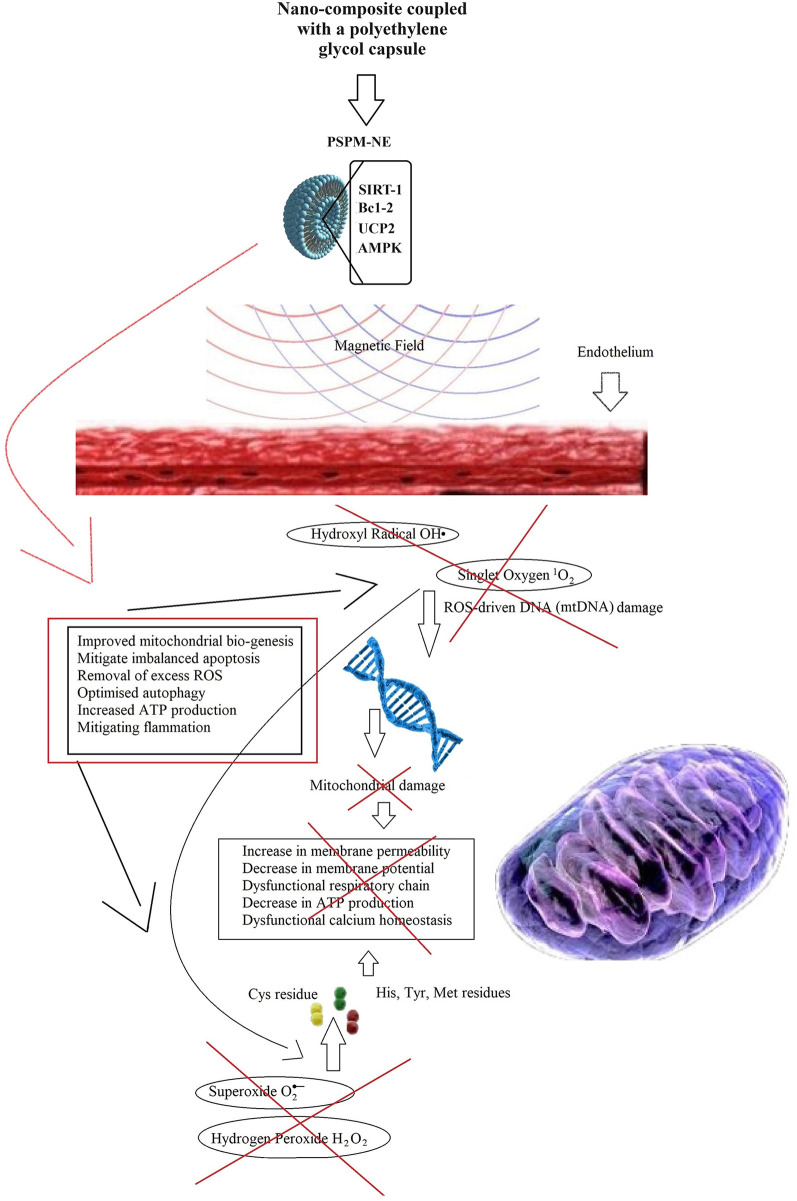
ROS-reduction and progressive cellular damage attenuation with tissue regeneration induced by the PSPM-NE conjugate, treating atherosclerosis.

Therefore, the proposed PSPM-NE conjugate is a promising ROS-regulating agent, mitigating apoptosis, reducing inflammation, and promoting tissue regeneration, in endotherial segments affected by atheroma (Figure 7).

Regarding potential side effects, it is important to assess the PSPM-NE conjugate under the perspective of cardio-toxicity, referring to the well-known QT prolonged interval as a biomarker.

The mechanism of cardiac depolarisation and repolarisation is cyclic. Depolarisation starts with an inward current of sodium ions (INa). INa is then inactivated, when the transient efflux of potassium ions (It0) occurs. An influx of calcium ions is then observed through L-type calcium channels (Ica), followed by an outward repolarising potassium currents (IK) called the plateau phase. Finally an efflux of potassium (IKr, IKu, IKs) and an inward of rectifier potassium current (IK1) are triggered to maintain resting potential.

The QT interval on an electrocardiogram (ECG) represents the duration of the action potential (AP) of ventricular myocytes, which represents the flow of ion currents across a cell membrane modulated by protein channels that help modulating the heart rhythm, i.e., the QT interval physiologically correlates with the duration of the ventricular depolarization and repolarization, and the AP potential reflects the electrical activity of cells.

When these protein channels are defective, inward currents may increase and outward currents may decrease, in a deadly manner. The related phenomena would result in an increase in the action potential duration. Hence, causing the QT interval prolongation. For this reason, the QT interval is a biomarker for severe cardiovascular dysfunction.

It shall be noted that an imbalance in the AP repolarisation may result from an increase or a decrease in an inward current, or alternatively from a decrease or an increase in an outward current (or eventually from alterations in both an inward and an outward current).

Cardio-toxicity may occur in many different ways. One important mechanism contributing to cardio-toxicity is the effect of drugs on the heart protein channels, which are therefore detected via alterations in the QT interval.

**Figure 8:**
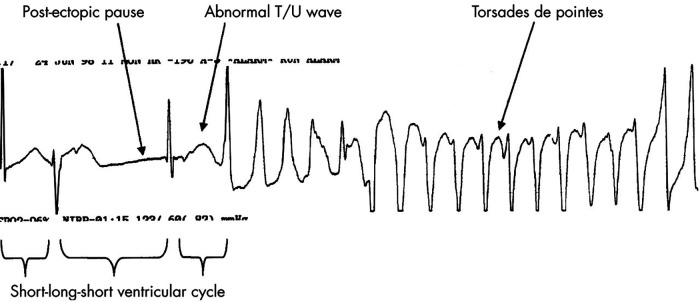
Rhythm strip in a patient with drug induced TdP. Note the typical short-long-short initiating ventricular cycle, pause dependent QT prolongation, and abnormal TU wave leading to the classical “twisting of a point” of the cardiac axis during TdP. Adapted from (82) Copyright © 2003 with permission from BMJ Publishing Group Ltd, https://doi.org/10.1136/heart.89.11.1363.

Among the diverse protein channels, the hERG channel is responsible for an important part of the cardiac depolarisation and repolarisation cycle. It represents the activity of the Kv11.1, which is the alpha sub-unit of this potassium ion channel, mediating the IKr rapidly activating delayed rectifier potassium current. If this potassium ion channel is defective, outward cell membrane current is inhibited or compromised. This may be caused by drugs or rare mutations. Mutation-driven impairment may result in a deadly disorder called long QT syndrome and in an inherited heart rhythm disorder named Short QT syndrome. Drug-driven QT irregularities may result in fatal ventricular tachy-arrhythmia (torsades de pointes, TdP). Figure 8 shows a generic illustration of torsades de pointes, commonly found in drug-induced QT irregularities. This ECG illustration showing TdP caused by drug-driven cardio-toxicity highlights a short-long-short pattern commonly seen in drug-induced TdP. Congenital-driven TdP episodes are usually linked to a sudden adrenergic (e.g., caused by exercise). Consecutive TdP episodes can progress to ventricular fibrillation and cardiac arrest.

Drug-induced toxicity resulting in a prolonged QT interval may occur via several mechanism. To mention a few, certain drugs used to treat gastroesophageal reflux (e.g., cisapride) may cause rescue of the SCN5A channel resulting in the increase of the inward sodium current. Antidepressants (e.g., fluoxetine), antimicrobials (e.g., pentamidine) and certain chemicals used in cancer treatment (e.g., arsenic oxide) may cause the disruption of KCNH2 protein trafficking affecting the K channels, resulting in a defective inward calcium current. An extensive list of drugs posing risk of prolonged QT interval can be found in the footnotes.^1^^,^^2^

In brief, the Polyphenolic Superparamagnetic Nano-Enzymatic Conjugate here proposed (PSPM-NE) minimises the risk of citotoxicity, more importantly, the risk of cardio-toxicity, by reducing off target interactions and by using encapsulated polyphenolic compounds showing negligible non-targetted effects.

The author proposes further studies, to completely quantify both the benefits and limitations of PSPM-NE when treating atherosclerosis.

## 3 Conclusions

In this manuscript, a novel drug design is presented, targetting both inflammatory and auto-immune signalling pathways associated with atherosclerosis initiation and progression. Signalling cascades and flow phenomena commonly used to characterise focal sites for atheroma development were assessed via both a haemorheological computational model of atherosclerosis and experimental findings on microfluidics under cell culture conditions. Together with findings from the current literature, this assessment supported selecting relevant inflammatory and auto-immune triggers, as the foundations for the proposed drug design. The correlation between drug-toxicity and QT interval prolongation formed the bases for selecting nanotechnological elements capable of mitigating drug toxicity, maximising drug concentration and effectiveness at target locations in the cardiovascular network, i.e., at focal sites for atheroma formation.

A nano-enzymatic conjugate combining SIRT-1 to stimulate mitochondrial bio-genesis, Bcl-2 to attenuate apoptosis, UCP2 to remove ROS, and AMPK to optimise autophagy was proposed. This nano-enzymatic plant active composite targets imbalanced ROS production and inflammation, mitigating mtDNA damages and mitochondrial protein alteration, to attenuate inter-cellular gaps caused by defective apoptosis and to stimulate mitochondrial function and ATP production. Altogether, to maintain a health endothelium, free of inflammation-driven atherosclerotic plaque deposition. This drug design is promising.

The design is enriched by a degradable polyethylene glycol superparamagnetic iron oxide capsule, resulting in a polyphenolic superparamagnetic nano-enzymatic (PSPM-NE) conjugate. The superparamagnetic capsule adds to the design the capability of being magnetically driven to focal sites of atheroma deposition, minimising off target interactions, maximising drug concentration at target locations, reducing the risk of toxicity.

Initial results suggest that PSPM-NE may be a good candidate for new therapeutics in atherosclerosis research. However, vast research is needed to further elucidate the capabilities of this new therapeutic design in clinics.

The author proposes further studies, to completely quantify both the benefits and limitations of PSPM-NE in atheroma treatment (i.e., its effects on atheroma biomarkers), which could then justify proposing future translational and clinical studies.

Finally, in this research project, the author observed that if the imbalance in ROS production is a major villain in cellular deterioration, with severe consequences in adult organisms whose cell proliferation potential is reduced, combined with defective autophagy, then, one may argue that together with tissue regeneration techniques and genetic engineering, ROS-modulating mechanisms are the potential “cure” for aging and for numerous diseases whose initiation and progression are triggered by dysfunction caused by cellular deterioration and (or) auto-immune dysfunction.

## Data Availability

The original contributions presented in the study are included in the article/supplementary material. Further inquiries can be directed to the corresponding author.

## Footnotes

^1^ www.SADS.org

^2^ www.Torsades.org
